# Advancing virtual and hybrid team well-being through a job demand-resources lens

**DOI:** 10.1080/17482631.2025.2472460

**Published:** 2025-03-13

**Authors:** Cass Coulston, Sukhi Shergill, Ricardo Twumasi, Myanna Duncan

**Affiliations:** a Institute of Psychiatry, Psychology and Neuroscience, King’s College, London, UK; b Kent and Medway Medical School, Canterbury, UK

**Keywords:** Virtual teams, hybrid work, job demands resources, team well-being, leadership dynamics

## Abstract

As the modern workplace evolves, the shift to virtual and hybrid team working necessitates a re-evaluation of well-being. While workplace well-being research has predominantly focused on the individual level, understanding team-level well-being is critical, as its underlying psychological and social processes differ. This study applies the Job Demands-Resources (JD-R) framework to virtual and hybrid contexts globally, demonstrating the dual nature of demands and resources at the team level, where the same constructs may contribute to driving positive gain cycles or negative loss cycles of well-being. Through reflexive thematic analysis, we analysed thirty semi-structured interviews with leaders and twenty-nine focus groups with 3–6 team members each (*n* = 110) across more than twelve industries and geographies. Our findings revealed three candidate themes: “Choice Matters”, “It’s Business and It’s Personal” and “Leader as Social Influencer”. This research extends JD-R theory by advancing its applicability to team-level well-being in virtual and hybrid contexts. Practical insights include empowering teams through redesigning work practices to establish sustainable boundaries, aligning communication norms, and fostering inclusive connections that accommodate diverse needs in the modern workplace.

## Introduction

The COVID-19 pandemic transformed the landscape of work, particularly in knowledge-based industries. Accelerated by globalization, digitalization, and technological progress (Lunde et al., 2022; Raghuram et al., 2019), the global adoption of virtual and hybrid working practices sparked a significant re-evaluation of how, when and where work is conducted. This shift has driven the rapid adoption of new approaches for collaboration, coordination and connection within teams. According to the Chartered Institute of Personnel and Development (CIPD, 2022), more than 57% of organizations have adopted hybrid working models, integrating office and remote working days into the weekly schedule. Identified as one of the most significant trends likely to shape the next two decades (Robinson et al., 2022), hybrid work profoundly influences team dynamics and well-being.

Despite its widespread adoption, the literature on virtual and hybrid work remains inconclusive (Grobelny, 2023), producing mixed findings on its implications for team well-being. Poor well-being has broad consequences, including increased mental health costs, burnout, absenteeism, and reduced effectiveness. Yet, workplace well-being research has disproportionately focused on the individual level (Bliese et al., 2017; Charalampous et al., 2019), overlooking interdependencies within teams. Well-being at the individual level is not independent; it is shaped by leadership, team interactions and collective attitudes (Jiang & Probst, 2016; Liu et al., 2015). This highlights the urgent need to better understand team-level processes that shape both individual and collective well-being.

This study responds to calls for a broader perspective in work-related well-being research (Kozusznik et al., 2015), by investigating the underlying psychological and social processes within virtual and hybrid teams. Using qualitative methods, we examine the experiences of team members and leaders working in virtual and hybrid settings. We discuss the implications for team well-being and organizational sustainability. The findings provide actionable insights for fostering healthier, more inclusive work environments, while also identifying limitations and areas for future research.

## Research objectives

The primary aim of this exploratory study was to enhance our understanding of the most salient well-being factors of virtual and hybrid teams, through a qualitative organizational investigation. This investigation examined these phenomena at both individual and team levels, synthesizing perspectives from team members and their leaders across diverse contexts. Specifically, this study contributes to organizational psychology and the health and well-being literature in several ways.

Firstly, the research employed reflexive thematic analysis (Braun & Clarke, 2006, 2019; Clarke & Braun, 2017), to ensure methodological rigour. This systematic approach facilitated the identification of the most salient themes, grounding findings in the participants’ voices and experiences to provide a nuanced understanding of well-being. Secondly, this study was theoretically rooted in the Job-Demands Resources (JD-R) theory (Bakker & Demerouti, 2007). By extending this framework to virtual and hybrid teams (Demerouti & Bakker, 2022; Li et al., 2022), the study provided a multi-level perspective on the dual nature of demands and resources, highlighting their potential role in driving both positive gain cycles and negative loss cycles of well-being (Bakker et al., 2023). Thirdly, this research emphasized the complex and context-dependent nature of team well-being, shaped by the dynamic interaction of individual, team, and leadership processes. Using a multi-level lens, this study aimed to uncover how these factors interact to influence team well-being in virtual and hybrid environments. Finally, this study provides actionable insights for organizations and policymakers. By addressing underexplored areas in the literature on team well-being (Bakker et al., 2023; Grobelny, 2023) this research offers timely contributions to both academic understanding and practical applications in the evolving field of hybrid work.

## Theoretical grounding

### A job demands-resources perspective on team well-being

This study is grounded in the Job Demands-Resources theory (JD-R) (Bakker & Demerouti, 2007), a well-established framework for understanding workplace well-being. Within the JD-R framework, job demands encompass physical, social, or organizational facets of the job that necessitate sustained physical, emotional, or mental effort. These demands are associated with physiological and psychological costs that individuals may incur (Demerouti et al., 2001). On the other hand, job resources encompass those aspects of the job that serve various benefits, such as facilitating work goal attainment, alleviating job demands and their associated costs and fostering personal growth and development (Demerouti et al., 2001). The JD-R theory posits that elevated job demands can result in strain and health-related challenges, while job resources correlate with enhanced motivation, heightened productivity, and improved well-being (Schaufeli & Taris, 2014). The JD-R framework provided a versatile lens to analyse the multi-faceted dynamics of well-being in hybrid and virtual teams. Unlike prescriptive models that narrowly specify factors for investigation, the JD-R model’s adaptability allowed this study to explore how demands and resources interact in diverse, context-dependent ways.

Although the JD-R framework offers a robust theoretical foundation, it is not without limitations. Its heuristic nature, while enhancing adaptability, has been critiqued for lacking explanatory depth regarding the psychological mechanisms that underpin relationships between demands, resources, and outcomes (Schaufeli & Taris, 2014). Additionally, despite the prevalence of teams, only few studies have utilized the team as the unit of analysis (Li et al., 2022).

By situating the JD-R model within a qualitative, multi-level study of virtual and hybrid teams, this research addresses these limitations and extends the model’s applicability. Specifically, it explores how team well-being is shaped by the interaction of individual, team, and leadership processes, providing deep insights into the dynamic nature of demands and resources in virtual and hybrid teams. This enhances the JD-R framework’s ability to capture the complexity of collective well-being (Bakker et al., 2023), contributing to both theoretical advancement and practical applications for supporting virtual and hybrid teams.

## Defining well-being

Work-related well-being incorporates various dimensions in the literature (Simone, 2014). According to (Fisher, 2014), well-being is composed of three distinct factors: hedonic, eudaimonic, and social well-being. Firstly, in a work context, hedonic well-being suggests a satisfying work life, involving a harmonious combination of positive affect, such as enthusiasm and inspiration and the absence of negative affect, such as stress and frustration (Fisher, 2014). Secondly, eudaimonic well-being encapsulates the fundamental human need for competence, autonomy, and relatedness (Fisher, 2014; Ryan & Deci, 2001; Ryff, 2014), contributing to a sense of purpose and fulfilment within the work context. Thirdly, social well-being focuses on the quality of connections and relationships within the workplace that can create contentment with peers, satisfactory exchange relationships with leaders, and the presence of social support and feelings of belonging (Simone, 2014). Collectively, these dimensions provide a holistic framework for understanding distinct factors of influence of work-related well-being, which is the multi-dimensional lens encapsulated for the current study.

## Method

Qualitative research as the methodological approach for this study stems from its unique capacity to unveil insights by exploring subjective experiences that may be challenging to capture through conventional quantitative methods (Hefferon et al., 2017). This approach enabled us to explore contextualized lived experiences within the broader social context, providing a deeper understanding of how well-being was shaped multi-dimensionally by the dynamics of the working environment. By embracing the complexity of participants’ experiences, we sought to move beyond surface-level observations and instead captured the depth and complexity of their lived experiences. Thus, we employed both qualitative semi-structured interviews and focus groups for the purpose of this study to gain this detailed understanding. The study received full ethical approval by the PNM Research Ethics Panel, King’s College London, (Ref: LRS/DP-21/22–26093). All data was collected between the 11th November 2021–16 March 2022.

## Participants

To gain insight from teams across a diverse range of industries, sectors, and geographies, our inclusion criteria were:

- Up to four teams from any organization utilizing virtual *and* hybrid working, to ensure we captured a diversity of teams.
- Participants aged 18+ years, working at least two days a week remotely from the office as part of a defined team.
- Within each team, a minimum of three team members were required for each 60-minute focus group, to ensure we were interviewing a group from a team. This excluded the team leader.
- All participants to have fluency in English and be able to participate via the social networking site of Zoom or Teams.

Twenty-nine teams (*n* = 110 team members) and thirty team leaders were recruited from both the public and private sector, capturing the voice of 140 participants globally. The team leader of each team focus group was interviewed in a separate 60-minute semi-structured interview to capture insights from the leader’s perspective. Recruitment was facilitated by our industry partner as well as the research team’s network, recruiting teams from across a range of different industries and geographies. An invitation to participate was sent via email along with an information sheet and consent form. Study participation was voluntary, as was withdrawal from the study (up to three months after participating) with participants providing digital consent.

## Data availability statement

Anonymized data of all transcriptions can be made available upon direct contact with the authors of this study.

Tables I and II provide a detailed overview of participant demographics. Participants were drawn from nine different private sector industries, including Banking (23%), Professional services (17%), Financial services (13%), Consultancy (10%), as well as public sector Education (10%), Healthcare (7%) and Not for Profit (7%). Roles spanned multiple levels, encompassing team members in operational, administrative, managerial and director roles as well as team leaders from Manager to Managing Director, enabling a comprehensive exploration of well-being across hierarchical levels and job functions.

**Table I.** Demographics of team leaders.

Team leader	Gender	Age	Ethnicity	Role within Organisation	Location of Leader	Industry	Number of days/week usually working virtually at time of interview
1	Female	35–44	White	Senior Manager	United Kingdom	Education	3 to 4
2	Female	35–44	White	Director	United Kingdom	Banking	3
3	Female	35–44	White	Director	Ireland	Professional services	3 to 4
4	Female	25–34	Arabic	Senior Manager	United Kingdom	Healthcare	4 to 5
5	Male	35–44	Asian	Senior Manager	India	Professional services	5
6	Male	45–54	White	Executive	Netherlands	Professional services	2 to 2 1/2
7	Male	45–54	White	Director	South Africa	Consultancy	2 to 3
8	Male	45–54	White	Senior Manager	United Kingdom	Education	3
9	Female	35–44	White	Director	United Kingdom	Professional services	4 to 5
10	Female	45–54	White	Senior Manager	Italy	Human Resources	3
11	Female	35–44	White	Senior Manager	Netherlands	Consultancy	5
12	Female	45–54	White	Executive	United Kingdom	Not for Profit	1
13	Female	45–54	White	Director	United Kingdom	Healthcare	4 to 5
14	Female	45–54	White	Director	United Kingdom	Technology	4 to 5
15	Female	25–34	White	Executive	Europe/Caribbean	Not for Profit	4 to 5
16	Female	35–44	White	Manager	United Kingdom	Education	4
17	Female	45–54	White	Senior Manager	Sweden	Human Resources	5
18	Male	45–54	White	Managing Director	United Kingdom	Banking	2 to 3
19	Female	25–34	White	Manager	United States	Consultancy	4
20	Female	35–44	White	Director	United States	Consultancy	4 to 5
21	Male	45–54	White	Executive	France	Professional services	2 to 3
22	Male	45–54	White	Manager	United Kingdom	Banking	4 to 5
23	Female	35–44	Asian	Senior Manager	China	Banking	2 to 3
24	Male	45–54	White	Managing Director	United Kingdom	Banking	5
25	Male	45–54	Asian	Managing Director	India	Banking	5
26	Male	35–44	White	Managing Director	Hong Kong	Banking	mixed
27	Female	35–44	White	Senior Manager	Spain	Financial services	2 to 3
28	Female	25–34	White	Manager	United Kingdom	Financial services	5
29	Male	55–64	White	Executive	Australia	Financial services	2 to 3
30	Female	45–54	White	Director	United States	Financial services	mixed

**Table II.** Demographics of teams.

Team	Number of Team members in focus group	of which male	of which female	Location of Team	Industry	Number of days/week usually working virtually across team at time of inteview
1	3	0	3	United Kingdom	Education	3 to 4
2	3	1	2	Global	Banking	5
3	3	1	2	Ireland	Professional services	4 to 5
4	3	1	2	United Kingdom	Healthcare	4 to 5
5	4	3	1	India	Professional services	4 to 5
6	4	3	1	Netherlands	Professional services	3 to 5
7	3	0	3	South Africa	Consultancy	4 to 5
8	3	1	2	United Kingdom	Education	3 to 5
9	4	2	2	United Kingdom	Professional services	3 to 5
10	4	3	1	Europe	Human Resources	2 to 5
11	4	2	2	Netherlands	Consultancy	3 to 4
12	4	0	4	United Kingdom	Not for Profit	2 to 3
13	3	0	3	United Kingdom	Healthcare	4
14	3	1	2	United Kingdom	Technology	4 to 5
15	4	1	3	Global	Not for Profit	4 to 5
16	4	1	3	United Kingdom	Education	0 to 4
17				no data		
18	5	4	1	United Kingdom	Banking	3
19	4	2	2	United States	Consultancy	4 to 5
20	5	2	3	United States	Consultancy	4 to 5
21	3	3	0	France	Professional services	3 to 4
22	6	3	3	United Kingdom	Banking	3 to 4
23	4	0	4	China	Banking	2
24	4	2	2	Global	Banking	2 to 4
25	3	1	2	India	Banking	5
26	4	3	1	Hong Kong	Banking	4 to 5
27	5	4	1	Spain	Financial services	4 to 5
28	3	1	2	United Kingdom	Financial services	5
29	4	3	1	Global	Financial services	1 to 5
30	4	2	2	United States	Financial services	5
Total	110	50	60			

Geographically, the study included participants from over thirteen countries, with 38% of teams from the UK, 21% from across Europe, 14% from Asia, 14% from global locations, as well as 10% from the United States and 3% from South Africa.

## Procedure

This qualitative design entailed sixty-minute virtual focus groups involving team members from the same team and separate sixty-minute semi-structured interviews with the team leader of the team. Both the focus groups and interviews were conducted via widely accessible virtual platforms; Microsoft Teams and Zoom. This approach was particularly suited to the research context, as participants were already accustomed to using these technologies for professional collaboration. Once recorded, all sessions were transcribed verbatim and verified for accuracy, after which the recordings were deleted. To guide the interviews, an interview guide was developed (see Supplementary files), incorporating open-ended questions that encouraged participants to share their thoughts, feelings, and perspectives. The questions were piloted in two sessions (one with team members and one with team leaders) to ensure clarity and relevance of questions. While no significant adaptations were required beyond the virtual format, the use of online platforms offered several benefits, including ease of scheduling, accessibility for participants across various locations, and the ability to foster an inclusive environment where participants could contribute comfortably from their own environment. For teams, examples of open-ended questions included:

- What does team well-being mean to you?
- Could you share any examples of well-being practices you have as a team that help your levels of well-being? Anything that gets in the way?
- What would you say are some of the biggest demands you face as a team that can lead to stress? What support could you benefit from?

For leaders, example questions included:

- What does work-related well-being mean to you?
- What would you say are some of the biggest demands you face as a leader that can lead to stress?
- How important is how you communicate as a team to your well-being?

## Analysis

**Figure 1.**
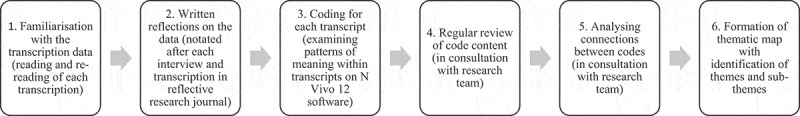
Thematic analysis six-stage process in accordance with (Braun & Clarke, 2006).

All interviews were transcribed verbatim, either by an external data transcription (*n* = 25) service or by a member of the research team (*n* = 34). Any identifiable data (e.g., names, organizational information) was removed to safeguard confidentiality of all participants and their organizations. NVivo12 software (QSR, 2018) was employed for data organization and management. Reflective thematic analysis (Braun & Clarke, 2006, 2019; Clarke & Braun, 2017), facilitated a predominantly inductive in-depth exploration, underpinned by critical realist assumptions, as outlined in Figure 1.

In accordance with the suggested six-stage analysis process, codes were generated from the data, developed, and reviewed for each transcript (Braun & Clarke, 2006; Clarke & Braun, 2017). The research team engaged in collaborative discussions to identify and name sub-themes and candidate themes, meticulously reviewing the coded data for each theme and refining coding structures where relevant. This rigorous and reflexive process ensured coherent patterns were identified and themes remained faithful to the data.

## Reflexivity statement

This study was developed by main author (Cass Coulston), as part of a postgraduate research programme focused on factors influencing the well-being and performance of virtual and hybrid teams. This part of the design process was to yield insights directly from team members and leaders that would help to inform future quantitative research into the same phenomena. Cass has had an interest in this topic for many years, having been a practitioner within organizations, and having worked as part of a team and leading a team.

Cass Coulston has had direct supervision from Dr Myanna Duncan and Dr Ricardo Twumasi through the process, who also have an interest in well-being and have previously published research on workplace stress and interventions to improve well-being at work. Professor Sukhi Shergill has published extensive research on social cognition, understanding the factors influencing social interaction, and impacts on mental health.

## Results

**Figure 2.**
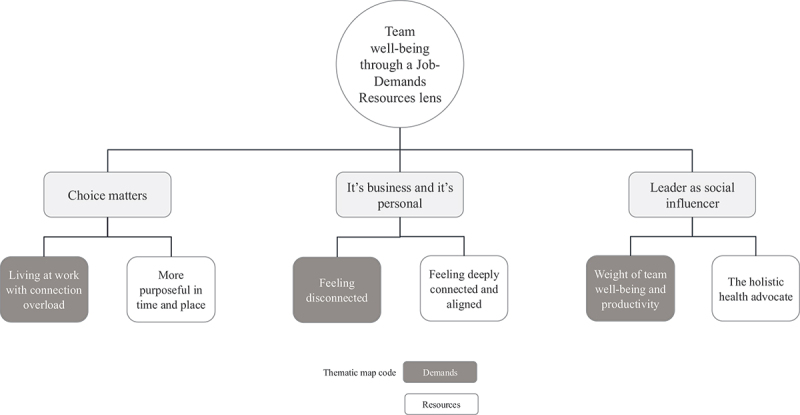
Thematic map of candidate and sub-themes.

The analysis of the interview transcripts led to the identification of three candidate themes, relevant for both team members and their leaders, which were elaborated on through the identification of six sub-themes. A visual representation of this thematic map is illustrated in Figure 2. A summary table of results (Table III) provides a further overview of the themes, sub-themes together with illustrative quotes. In the subsequent result sections of this paper, an exploration of the identified themes and sub-themes is provided, complemented by relevant participant quotations. The viewpoints shared by both leaders and team members are integrated, with tensions highlighted, where appropriate.

**Table III.** Summary table of themes, sub-themes and illustrative quotes.

Candidate Themes	Sub-Themes	Illustrative Quotes
Choice Matters	Living at work with Connection Overload	*“You could spend your days in back-to-back meetings. I don’t even have the time to break for lunch or quite simply for work, to rest, for your mind to get some sleep. I can see more and more clients. I had one this morning telling me: Well, I’m available at 07:30 this morning, so let’s have a quick call. Oh, I’m available tonight at 19:00… . So, I think this is changing a lot.”* Team leader, Team 21, Professional Services, France. *“What I’m seeing is that there is an abuse of over-communicating from my perspective. Everyone needs to know everything, which is not really working in this for me. It’s not really working in this context, because we’re just overwhelming each other with too many communications and people are not reading*.” Team leader, Team 10, Human Resources, Europe. *“You can say well-being is important. You can say that you need to take care of yourself. You can say that you need to have work life balance. But if you’re looking and you’re seeing partners are on 24/7, they’re never off, never eating. Yesterday I was talking to a leader and for dinner they were having nuts and cheese, and so if that’s what they’re doing, you feel guilty about taking time for yourself and taking care of yourself.”* Team member 5, Team 19, Consultancy, US.
	More Purposeful in time and place	“*I think the pandemic has been a big wake up call for many of us. We have got, you know, a better taste, a better balance of how we actually neglected our self-care earlier, you know? So, now, for example, if I have to attend some yoga class or some gym class, whatever, I can, I simply put it in my calendar and block one hour for myself. And, you know when I’m in the virtual environment, I can attend that. So yeah, we have got our taste of better balance now, and we’re not ready to give it up.”* Team member 1, Team 25, Banking, India. *“I’ve reinvented the wheel of family life. I was maybe two, three days a week that I joined dinner and for the rest of week, I wasn’t there simply. Now I’m there at least four or five days a week out of seven. So that’s super improved. I’m also now a trainer on the field hockey team of my son, where I couldn’t believe I would do that at evening time at half past five. So that really changed from a professional well-being sphere. I also enjoy a lot that I see colleagues also changing their lifestyle*.” Team Leader, Team 6, Professional Services, Netherlands. *“There is like an allowance of time per week … it works out about half an hour a day, where you could just do something that’s not work related … you could do some yoga, or you could just go for a walk in that time, or it might mean going for a run at lunchtime. I used to try and do everything in an hour … and I’ll just be at my desk being completely exhausted. Whereas now, I just know that if I take a bit longer than an hour doing all of that then that’s covered if you like by the well-being allowance.”* Team member 2, Team 15, Not for Profit, Global.
It’s business and it’s personal	Feeling disconnected	*“I think it’s easy to feel like your world is contracted … so you might work in a company that has a very broad, diverse range of people, but definitely the world contracts when the people that you chat to reduces, is limited over a long period of time.”* Team member 3, Team 14, Technology, UK. *“I think about my work and my happiness at work as like a sphere, as a ball made up of all different stuff floating around, it’s a little mini universe. There’s a huge hole there now for me, where that true team connect, you know, that instant kind of gratification, feedback, understanding, partnership, all of that stuff, it’s missing.”* Team member 1, Team 10, Human Resources, Europe. *“You are more remote from these people and you’re never quite sure how someone is really feeling and then just in the same way as if you’re in the office, you can read body language, or the people are likely to more open up to you because they don’t have to go through the positive effort of… . you can just have a chat with them.”* Team leader, Team 26, Banking, Hong Kong.
	Feeling deeply connected and aligned	*“I think being able to make time to connect with each other just as people, both like setting aside special time or you know particular time to do that, as well as just doing that whenever we are on zoom together. I don’t really know how to define it, but I don’t feel like we would have team well-being if we didn’t do that. If all we did was jump into the work, it wouldn’t feel like we were really a team. It would feel like we were just individuals, updating each other.”* Team member 1, Team 15, NGO, Global. *“So creating that sense of it’s a place where I feel cared for, I feel there’s empathy. People understand, appreciate where I’m at and there’s space to share personally. And I’ve seen that’s a very good practice. Sometimes, I desire sometimes just to do that. Let’s talk about the numbers, the results. Let’s track them. And we end up spending half an hour people just checking in and talking about how it’s going with the family and this thing happened with the dog. But I think those moments are very important.”* Team Leader, Team 7, South Africa. *“We’ve also been doing this now, I think, for a good year, where once a month, one member of the team just speaks about themselves… . and I think it just allowed everyone to feel a little it more open and to [name]‘s point, senior management started it, so it sort of removes that layer of they’re higher than me and there’s a bit of a difference between us, because we’ve gotten to know them. They’ve told us about their personal lives, they’ve been quite open about it… . . I think it really removes all the layers of work and feeling drained.” Team member 2, Team 24, Banking, Global.*
Leader as social influencer	Weight of team well-being and productivity	*“I’ve had quite a few of the team with various versions of burnout I guess, and I found it really, really hard to see. I found that quite emotionally taxing to see people in that state and in that position, and obviously you never want to see your team struggle or be unhappy. And so yeah, I’ve, I found that very difficult and very worrying.”* Team leader, Team 14, Technology, UK. *“I feel the responsibility of the whole team… . So I always think—I know what I can do. So I can manage myself. But I cannot manage the feelings of 20 people. So that’s why I’m more concerned about the others and making sure that they are ok and trying to meet their needs, because I know myself how to handle myself. Maybe I shouldn’t. Maybe I’m sacrificing myself over the others.”* Team leader, Team 1, Education, UK. *“When you’re sitting in the office, you observe more … And at home for example, I’m not hearing it, but when I’m in the office and they’re in their office, I do pick up stuff. So whereas when you’re working remotely, you can only see what they actually let you see and what they tell you. Not that they’re trying to hide anything, but it’s natural, right? Whereas when they’re in the office, you get to know the staff more. And you see how they behave, and you can coach them a bit more.”* Team leader, Team 27, Financial Services, Spain.
	The holistic health advocate	*“I think our team lead also plays a huge role in facilitating the focus on the human and checking in on each other. Whenever we check in as a team, she always takes the lead on first, how’s everybody doing, what have you been up to? How are you feeling? and really creating a safe space to actually share what’s going on as opposed to the good news stories, so to speak.” Team member 1, Team 11, Consultancy, Netherlands*. *“I think if I don’t see the leaders on my project, taking breaks to go and walk during the day, or do things like that, I’m less inclined to do that because I think I have to be on. So you know, by seeing them set examples like that and really follow through, then I feel more inclined to and empowered to do those things… And I feel comfortable to do that because this truly is a team and a leadership team that values that.” Team member 1, Team 19, Consultancy, US*. *“I mean, you can ask someone what they’re doing just to blow the time away and fill up the first few minutes or you can try and work out what they’re doing or what they’re up to to try and work out well, how can we help you, … why don’t you take that afternoon off or why don’t you do this ‘cause, I think it’s important … . And people caring about you and then you’ve got flexibility to offer workers. But what do they want? What out of all the lists of things you know have we got that we can offer them that’s meaningful and how do we have the meaningful conversation … I think it’s got to matter. “Team leader, Team 29, Financial Services, Australia.*

## Theme one: choice matters

This overarching theme, “Choice Matters,” reflects how autonomy and decision-making processes profoundly influenced the well-being of team members and leaders. The influence of choice, or conversely, the perception of a lack of choice, was identified, with team members and their leaders making daily choices that exerted a considerable influence on their well-being outcomes. Participants navigated a fine balance between demands that left them feeling powerless and resources that fostered a sense of purposefulness. Two sub-themes, “Living at Work with Connection Overload” and “More Purposeful in Time and Place,” illustrated how the dynamics of virtual and hybrid work shaped these experiences.

### Sub-theme one: living at work with connection overload

For many team members and their leaders, the absence of clear boundaries between starting and ending the workday led to feelings of overwhelm and stress, most notably when working from home. Work from home often extended the workday, as time once allocated for commuting, and for some, decompression time, was absorbed into back-to-back meetings. leading to cognitive and emotional challenges for participants to switch off.

You could spend your days in back-to-back meetings. I don’t even have the time to break for lunch or quite simply for work, to rest, for your mind to get some sleep. I can see more and more clients. I had one this morning telling me: Well, I’m available at 07:30 this morning, so let’s have a quick call. Oh, I’m available tonight at 19:00…. So, I think this is changing a lot. (Team leader, Team 21, Professional Services, France)

The overwhelming pace of communication exacerbated this issue. The resulting connection overload led to feelings of exhaustion and anxiety, ineffective multi-tasking, with some likening their experience to facing a “tsunami of emails” or a “barrage” of messages.

What I’m seeing is that there is an abuse of over-communicating from my perspective. Everyone needs to know everything, which is not really working in this for me. It’s not really working in this context, because we’re just overwhelming each other with too many communications and people are not reading. (Team leader, Team 10, Human Resources, Europe).

Participants described a relentless need to stay connected across multiple platforms, often driven by a combination of self-imposed internal assumptions of needing to appear reliable and productive and fears of not being seen or recognized, as well as external expectations, often modelled by leaders’ own behaviour of communication.

You can say well-being is important. You can say that you need to take care of yourself. You can say that you need to have work life balance. But if you’re looking and you’re seeing partners are on 24/7, they’re never off, never eating. Yesterday I was talking to a leader and for dinner they were having nuts and cheese, and so if that’s what they’re doing, you feel guilty about taking time for yourself and taking care of yourself. (Team member 5, Team 19, Consultancy, US)

Despite recognizing the importance of self-care, participants often struggled to implement meaningful boundaries and lifestyles had become more sedentary for some. This sense of powerlessness was further compounded by a perceived need to overcompensate for home-based distractions, particularly for those managing childcare responsibilities.

### Sub-theme two: more purposeful in time and place

In contrast, several leaders and team members experienced hybrid work as an opportunity to make more intentional choices about how, when, and where they worked. This sense of autonomy acted as a powerful resource, enabling participants to align their professional responsibilities with personal goals. Words like “flexibility,” “freedom,” “balance” and “empowerment” were frequently used to describe these experiences.

I think the pandemic has been a big wake up call for many of us. We have got, you know, a better taste, a better balance of how we actually neglected our self-care earlier, you know? So, now, for example, if I have to attend some yoga class or some gym class, whatever, I can, I simply put it in my calendar and block one hour for myself. And, you know when I’m in the virtual environment, I can attend that. So yeah, we have got our taste of better balance now, and we’re not ready to give it up. (Team member 1, Team 25, Banking, India)

Participants emphasized that hybrid work offered new opportunities to manage their time in ways that fostered well-being. For some, the office became a space for social interaction and collaboration, while home provided an environment for focused, independent work. This approach was aspirational for many but offered new opportunities to integrate their personal and professional working lives.

I’ve reinvented the wheel of family life. I was maybe two, three days a week that I joined dinner and for the rest of week, I wasn’t there simply. Now I’m there at least four or five days a week out of seven. So that’s super improved. I’m also now a trainer on the field hockey team of my son, where I couldn’t believe I would do that at evening time at half past five. So that really changed from a professional well-being sphere. I also enjoy a lot that I see colleagues also changing their lifestyle. (Team Leader, Team 6, Professional Services, Netherlands)

Effective communication and openness among team members was seen as key, as participants recognized the need to acknowledge and respect each other’s time, energy, and differing needs. These purposeful choices often translated into practical strategies, such as adopting “well-being hours.” This autonomy not only empowered individuals but also fostered stronger connections within teams, reinforcing the importance of aligning work practices with well-being goals.

There is like an allowance of time per week … it works out about half an hour a day, where you could just do something that’s not work related … you could do some yoga, or you could just go for a walk in that time, or it might mean going for a run at lunchtime. I used to try and do everything in an hour… and I’ll just be at my desk being completely exhausted. Whereas now, I just know that if I take a bit longer than an hour doing all of that then that’s covered if you like by the well-being allowance. (Team member 2, Team 15, Not for Profit, Global)

## Theme two: It’s business and it’s personal

This overarching theme captured the complex and evolving interaction between work and personal connectivity in virtual and hybrid environments. Participants described how the right balance could foster a sense of resourcefulness and fulfilment, while misalignment created distress. The two sub-themes, “Feeling Disconnected” and “Feeling Deeply Connected and Aligned,” illustrated the contrasting experiences of isolation and connection in virtual and hybrid teams.

### Sub-theme 1: feeling disconnected

I think it’s easy to feel like your world is contracted … so you might work in a company that has a very broad, diverse range of people, but definitely the world contracts when the people that you chat to reduces, is limited over a long period of time. (Team member 3, Team 14, Technology, UK)

For many participants, virtual and hybrid working heightened feelings of disconnection, with approximately half describing challenges in maintaining the emotional and relational bonds they associated with co-located work. Participants acknowledged that the spontaneity and authenticity of office interactions were difficult to replicate virtually, where communication often felt transactional. Virtual settings also reduced the ability to observe body language and other non-verbal cues, making it harder to gauge motivation or emotional states, with limited opportunities for spontaneous support, which participants described as integral to team well-being in physical office settings.

I think about my work and my happiness at work as like a sphere, as a ball made up of all different stuff floating around, it’s a little mini universe. There’s a huge hole there now for me, where that true team connect, you know, that instant kind of gratification, feedback, understanding, partnership, all of that stuff, it’s missing. (Team member 1, Team 10, Human Resources, Europe)

Informal camaraderie and bonding time within some teams appeared to be more challenging to create when working remotely. Consequently, opportunities for building trust and understanding were not seen as readily available, leading to a sense that connections needed to be orchestrated and could sometimes feel artificial. Participants working in isolation for prolonged periods also described reduced opportunities for learning and collaboration, which led to feelings of anxiety, self-doubt, and fears of being forgotten. This led to a view from some team members and leaders that people were working in a more siloed, individualistic way.

You are more remote from these people and you’re never quite sure how someone is really feeling and then just in the same way as if you’re in the office, you can read body language, or the people are likely to more open up to you because they don’t have to go through the positive effort of… . you can just have a chat with them. (Team leader, Team 26, Banking, Hong Kong)

### Sub-theme two: feeling deeply connected and aligned

In contrast, participants who experienced meaningful connection within their teams emphasized the intentionality required to foster this resource. Both leaders and team members noted that creating a sense of deep connection was pivotal for enhancing well-being, engagement, and work effectiveness. Many highlighted the importance of cultivating trust and open communication.

I think being able to make time to connect with each other just as people, both like setting aside special time or you know particular time to do that, as well as just doing that whenever we are on zoom together. I don’t really know how to define it, but I don’t feel like we would have team well-being if we didn’t do that. If all we did was jump into the work, it wouldn’t feel like we were really a team. It would feel like we were just individuals, updating each other. (Team member 1, Team 15, NGO, Global)

Intentional practices, such as regular check-ins or virtual social events, helped recreate moments of camaraderie. Activities like “fun Fridays,” quizzes, and informal team chats on platforms like Slack and WhatsApp were frequently cited as effective tools for building and maintaining team bonds. Leaders also played a crucial role in modelling these behaviours, particularly by sharing their own personal experiences and creating safe spaces for open dialogue.

So creating that sense of it’s a place where I feel cared for, I feel there’s empathy. People understand, appreciate where I’m at and there’s space to share personally. And I’ve seen that’s a very good practice. Sometimes, I desire sometimes just to do that. Let’s talk about the numbers, the results. Let’s track them. And we end up spending half an hour people just checking in and talking about how it’s going with the family and this thing happened with the dog. But I think those moments are very important. (Team Leader, Team 7, South Africa)

The ability to connect on a personal level often required shifting priorities away from purely task-oriented interactions. Many participants emphasized the significance of leaders fostering inclusive environments where every individual’s voice could be heard, even in virtual settings.

We’ve also been doing this now, I think, for a good year, where once a month, one member of the team just speaks about themselves … . and I think it just allowed everyone to feel a little bit more open and to [name]‘s point, senior management started it, so it sort of removes that layer of they’re higher than me and there’s a bit of a difference between us, because we’ve gotten to know them. They’ve told us about their personal lives, they’ve been quite open about it … . . I think it really removes all the layers of work and feeling drained. (Team member 2, Team 24, Banking, Global)

## Theme three: Leader as social influencer

This theme explored the systemic complexity of leadership in virtual and hybrid work contexts, identifying the pivotal role leaders played in shaping team well-being. Leadership was seen as both a resource that empowered teams and a demand that placed a heavy burden on leaders themselves. The two sub-themes, “Weight of team well-being and productivity” and “The holistic health advocate,” captured these contrasting dynamics, highlighting how leadership could either enhance or hinder team and leader well-being.

### Sub-theme one: weight of team well-being and productivity

I’ve had quite a few of the team with various versions of burnout I guess, and I found it really, really hard to see. I found that quite emotionally taxing to see people in that state and in that position, and obviously you never want to see your team struggle or be unhappy. And so yeah, I’ve, I found that very difficult and very worrying. (Team leader, Team 14, Technology, UK)

This sub-theme revealed the significant demands placed on leaders as they sought to navigate the demands of virtual and hybrid work. The absence of physical presence made it harder to perform informal “temperature checks,” leaving some leaders feeling uncertain and less in control of team dynamics. Many leaders described their struggles with maintaining real-time visibility into team activities and well-being.

The pressure of responsibility weighed heavily on leaders, particularly when team members’ well-being seemed intertwined with their own. Leaders described sleepless nights and heightened stress levels, often feeling stretched thin between balancing team needs and their personal well-being.

I feel the responsibility of the whole team … . So I always think—I know what I can do. So I can manage myself. But I cannot manage the feelings of 20 people. So that’s why I’m more concerned about the others and making sure that they are ok and trying to meet their needs, because I know myself how to handle myself. Maybe I shouldn’t. Maybe I’m sacrificing myself over the others. (Team leader, Team 1, Education, UK)

For some leaders, their efforts to adapt led to over-communication. The challenge of balancing trust and accountability created ongoing tension, suggesting the need for leaders to adopt more creative communication strategies. Some leaders also expressed concerns that hybrid work had shifted relationships towards a more transactional nature, reducing the depth of connection and ability to engage team members.

When you’re sitting in the office, you observe more … And at home for example, I’m not hearing it, but when I’m in the office and they’re in their office, I do pick up stuff. So whereas when you’re working remotely, you can only see what they actually let you see and what they tell you. Not that they’re trying to hide anything, but it’s natural, right? Whereas when they’re in the office, you get to know the staff more. And you see how they behave, and you can coach them a bit more. (Team leader, Team 27, Financial Services, Spain)

### Sub-theme two: the holistic health advocate

In contrast, leaders who embraced a holistic health advocacy approach demonstrated how leadership could serve as a resource for team well-being. These leaders prioritized transparency and vulnerability, openly sharing their own well-being practices to inspire and empower their teams. By modelling self-care and balance, they fostered an environment of trust and inclusivity. They effectively “humanized” their leadership persona, empowering team members to prioritize and openly share their well-being journeys.

I think our team lead also plays a huge role in facilitating the focus on the human and checking in on each other. Whenever we check in as a team, she always takes the lead on first, how’s everybody doing, what have you been up to? How are you feeling? and really creating a safe space to actually share what’s going on as opposed to the good news stories, so to speak. (Team member 1, Team 11, Consultancy, Netherlands)

Practices like “walk and talks” and encouraging team members to integrate well-being activities into their schedules were frequently cited as sought after by team members. Team members expressed their gratitude for leaders who demonstrated a deeper understanding of individual team needs and role modelled healthy work practices themselves.

I think if I don’t see the leaders on my project, taking breaks to go and walk during the day, or do things like that, I’m less inclined to do that because I think I have to be on. So you know, by seeing them set examples like that and really follow through, then I feel more inclined to and empowered to do those things… And I feel comfortable to do that because this truly is a team and a leadership team that values that. (Team member 1, Team 19, Consultancy, US)

Leaders who cultivated safe spaces for open dialogue about well-being were particularly valued by their teams. These leaders emphasized care and empathy, recognizing and addressing the diverse needs of their team. They demonstrated how leadership could serve as a resource that enhanced both individual and collective well-being, illustrating a pronounced aspiration to cultivate environments where well-being assumed a central role.

I mean, you can ask someone what they’re doing just to blow the time away and fill up the first few minutes or you can try and work out what they’re doing or what they’re up to to try and work out well, how can we help you, knowing you’re doing that, why don’t you take that afternoon off or why don’t you do this ‘cause, I think it’s important …. And people caring about you and then you’ve got flexibility to offer workers. But what do they want? What out of all the lists of things you know have we got that we can offer them that’s meaningful and how do we have the meaningful conversation … I think it’s got to matter. (Team leader, Team 29, Financial Services, Australia)

## Discussion

The primary objective of this study was to move beyond existing well-being research by unpacking the multi-dimensional nature of well-being for virtual and hybrid teams. By exploring the lived experiences of team members and leaders (Braun & Clarke, 2006, 2021), and examining how job demands and resources interact dynamically, this study contributes to a deeper understanding of team well-being in contemporary work contexts. Grounded in the Job Demands-Resources (JD-R) model (Bakker & Demerouti, 2007), the findings demonstrate the relational and collective dimensions of well-being, highlighting how demands and resources are context-dependent and subject to variation based on team dynamics.

While the JD-R framework traditionally focuses on individual pathways of motivation and strain (Bakker et al., 2023), our findings highlight the value of incorporating team-level and relational processes to better capture the complexity of well-being in virtual and hybrid environments. Through thematic analysis, three salient themes “Choice Matters,” “It’s Business and It’s Personal,” and “Leader as Social Influencer,” highlighted the dual nature of demands and resources. Each theme illustrated how factors could simultaneously act as demands and resources, highlighting the complexity and non-linear nature of well-being in virtual and hybrid teams.

These findings also resonate with elements of Conservation of Resources (COR) theory (Hobfoll et al., 2018) particularly regarding resource accumulation and depletion. While this study’s cross-sectional design limits definitive conclusions about resource spirals, it suggests that virtual and hybrid work contexts may foster environments conducive to both resource gain and loss cycles. Participants who described intentional well-being practices, such as deliberate communication strategies and trust-building, appeared to experience resource accumulation, fostering resilience and engagement. Conversely, participants facing connection overload and blurred work-life boundaries reported resource depletion, which hindered their well-being.

The theme “Choice Matters” highlighted how autonomy and decision-making processes could function as both a resource and a demand, depending on the presence of enabling factors. As emergent literature suggests, hybrid work can offer advantageous resources in allowing participants to experience greater energy and well-being in their day (Tanpipat et al., 2021). For some participants, autonomy facilitated purposeful work-life integration, promoting resource gain. However, others found autonomy and greater choices burdensome when accompanied by heightened connectivity and a lack of clear boundaries. Participants who successfully made more purposeful choices often cited supportive leaders and well-defined team norms as critical enablers.

In line with previous studies, the findings demonstrate that work-life integration can serve as a key resource when individuals are empowered to manage their time and space flexibly (Feigon et al., 2018). Participants who utilized the flexibility of virtual and hybrid work to engage in personal pursuits, such as exercise or family activities, reported increased well-being. However, negative emotions such as guilt and anxiety led some participants to overcompensate, resulting in resource depletion and diminished well-being (Morrison-Smith & Ruiz, 2020). This highlights the need for structured support to prevent resource loss and foster resource gain in virtual and hybrid teams (Tanpipat et al., 2021).

The second theme, “It’s Business and It’s Personal,” demonstrated the significance of connection and relational dynamics in shaping well-being. The need-to-belong theory (Baumeister & Leary, 1995) posits that repeated social encounters are essential for emotional attachment and acceptance. This theory appears particularly relevant in virtual and hybrid work contexts, where uncertainty and isolation risks are heightened. Participants who felt deeply connected to their teams and leaders reported greater engagement, psychological safety, and well-being, corroborating research that links positive relational experiences with increased collaborative engagement and learning (Pope & Miles, 2022).

Conversely, participants who felt isolated or disconnected expressed higher levels of anxiety and disengagement. Emotional contagion (Hatfield et al., 1993) emerged as a key mechanism in these relational dynamics, with both positive and negative emotional states emerging through teams. As echoed in emerging research, teams that implemented intentional practices to foster connection, such as virtual “check-ins” and shared social activities, reported greater cohesion, teamwork transparency and well-being (Lechner & Tobias Mortlock, 2022; Methot et al., 2021; Morrison-Smith & Ruiz, 2020). In contrast, the absence of such practices led to negative emotional contagion, with stress and anxiety permeating teams. These findings suggest that relational resources are central to well-being in virtual and hybrid teams and emphasize the co-constructed nature of well-being through shared experiences and interactions (Lechner & Tobias Mortlock, 2022).

The third theme, “Leader as Social Influencer,” demonstrated the critical role of leadership in shaping team well-being. Leaders who modelled healthy behaviours, fostered trust, and created psychologically safe environments were viewed as pivotal resources by their teams. These leaders emphasized the deliberate effort required to balance productivity and well-being demands, highlighting the complexity and adaptability required to effectively lead in virtual and hybrid contexts (Cortellazzo et al., 2019; Hooijberg & Watkins, 2021). However, leaders themselves faced increased demands, with many reporting heightened stress and resource depletion (Poetz & Volmer, 2024) due to the dual pressures of maintaining team productivity and well-being.

The concept of emotional contagion (Hatfield et al., 1993) was particularly salient in this theme. Leaders who exhibited high stress levels often appeared to transmit these emotions to their teams, contributing to resource loss. Conversely, leaders who prioritized their own well-being and shared their strategies for maintaining balance fostered positive emotional contagion, promoting resource gain within their teams. This dynamic illustrates the cascading impact of leadership behaviours on team well-being.

Overall, this study extends the JD-R model by demonstrating how demands and resources operate not only at the individual level but also through team-level and relational processes (Bakker et al., 2023). The findings highlight the importance of context in shaping the dual nature of demands and resources in virtual and hybrid teams. By integrating insights from COR theory (Hobfoll et al., 2018), this research offers a new lens for understanding collective well-being, exploring the interaction of individual, team, and leadership processes in fostering resource accumulation and preventing resource depletion.

## Practical implications

This study provided valuable insights into factors influencing work-related well-being, operating at both individual and collective levels. Through reflexive thematic analysis, the findings offer a framework for organizations to implement targeted, actionable strategies aimed at improving team and leader well-being.

Within the theme of “Choice Matters,” we have highlighted the significance of deliberate time and space considerations for many team members, advocating for practices that facilitate a meaningful integration of professional responsibilities with personal well-being routines. Teams should undertake a critical evaluation of their communication practices, discerning both the timing and content of their interactions to foster purposeful communication strategies. This is supported by (Riedl & Woolley, 2017) who found that successful remote teams communicated in bursts interspersed with periods of individual focus, thereby enhancing productivity and reducing cognitive overload. Given the prevalent sense of being overwhelmed described by many teams in this study, organizations should reassess recurring communication modalities. Evaluations could replace less efficient modes, such as back-to-back lengthy video conferences, with concise alternatives like phone calls or asynchronous updates.

Furthermore, encouraging practices like “walk-and-talk” meetings or periods designated for focused work, such as “no-meeting hours” or “well-being hours,” were seen to help employees manage demands more effectively in this study. These strategies allowed team members to regain control over their schedules and prevent resource depletion caused by constant connectivity pressures.

Within the theme of “It’s Business and It’s Personal,” our research evidenced the importance of cultivating a strong sense of connection within teams, with structured opportunities for informal interaction playing a pivotal role. Intentional incorporation of unstructured time into the working week fostered genuine connection and rapport, contributing to increased psychological safety and engagement. Practices like virtual “check-ins,” social rituals, and informal conversations will help establish a supportive climate that promotes well-being (Pope & Miles, 2022). Moreover, the creation of team norms around social interactions (Lechner & Tobias Mortlock, 2022) can further enhance relational resources in virtual and hybrid teams.

To sustain these practices, organizations could consider developing “communication charters;” formalized guidelines that outline when and how team members communicate synchronously and asynchronously. This may help mitigate feelings of connection overload and promote more intentional interactions. Additionally, fostering inclusivity through initiatives that allow team members to voice preferences around their working patterns and communication methods can help create a more balanced and equitable work environment.

The theme “Leader as Social Influencer” highlights the central role leaders play in shaping team well-being. Leaders who modelled healthy behaviours and prioritized their own well-being were seen as key resources by their teams. Therefore, leadership training programs focused on well-being-centric practices, such as fostering psychological safety and setting healthy boundaries, would be beneficial (Poetz & Volmer, 2024). We would suggest incorporating leadership development that trains the importance of role modelling healthy behaviours, including taking regular breaks, maintaining boundaries between work and personal life, and encouraging open dialogue about well-being.

Furthermore, tailored coaching and mentoring programs could support leaders in managing the dual pressures of productivity and well-being. For instance, leaders who engage in “well-being conversations” with their teams can help identify potential stressors early and co-create solutions, distributing the responsibility for team well-being, thereby preventing burnout among leaders.

Finally, we recommend organizational interventions that combine short-term support with sustained structural and cultural changes. Short-term interventions, such as targeted well-being workshops, can provide immediate relief by addressing specific stressors. More sustained efforts could involve team-level redesigns that engage team members in co-creating their ways of working. For example, facilitated coaching workshops could guide teams in defining their shared values, work norms, and well-being goals, ensuring that both top-down and bottom-up perspectives are integrated. Such participatory approaches can enhance buy-in and foster a culture of collective well-being, recognizing that effective solutions must account for individual differences.

These practical implications reinforce the necessity of embedding well-being into organizational strategy, not as a peripheral initiative but as a core component of how teams operate. By actively engaging team members and leaders in these initiatives, organizations can create a sustainable environment where well-being is prioritized and protected.

## Limitations and future research

While this study provided valuable insights, it is essential to acknowledge its limitations, which offer opportunities for further exploration. Firstly, whilst interview guides were designed to elicit open-ended responses without leading participants, the inherent nature of qualitative inquiry means that responses may have been influenced by the interview context, including the presence of other team members in focus groups. This may have led to social desirability bias (Teh et al., 2023) with participants potentially displaying socially or culturally acceptable behaviours or downplaying negative experiences.

Secondly, the study predominantly included participants from private sector organizations, limiting the generalizability of the findings. The greater inclusion of industries such as healthcare, education, or public service sectors would provide a more comprehensive understanding of the unique demands and resources experienced in different work contexts. Additionally, the participant pool comprised individuals who were actively working and available at the time of data collection. Consequently, the experiences of those on extended leave, including sick leave or those experiencing burnout, were not captured. Including such voices in future research could offer critical insights into the more extreme ends of well-being, particularly regarding resource depletion and loss spirals.

Moreover, the cross-sectional nature of this study limits the ability to draw conclusions about the development of resource gain or loss spirals over time. While our findings indicate that virtual and hybrid team contexts may foster both positive and negative resource cycles, longitudinal research is required to examine these dynamics more rigorously. Specifically, longitudinal studies could track how resource dynamics evolve over time, exploring whether initial resource losses or gains influence longer-term outcomes in well-being and performance. Mixed-methods research could also be employed to identify moderators, such as team norms, leadership behaviours, and organizational culture, that may buffer against resource loss or enhance resource accumulation.

These findings also have important implications for the theoretical development of the JD-R framework (Bakker et al., 2023). By identifying relational and collective dimensions of well-being, our study suggests that the JD-R model would benefit from an expanded focus on team-level processes and leadership dynamics (Demerouti & Bakker, 2022). Integrating insights from the COR theory, particularly the asymmetrical impact of resource loss versus gain (Bakker et al., 2023; Hobfoll et al., 2018) could provide a more comprehensive explanatory model for understanding well-being in virtual and hybrid teams. Of particular interest is the assertion by Hobfoll et al. (2018) that resource loss exerts a disproportionately stronger impact than resource gain. Future mixed-methods research is needed to determine the extent to which this phenomenon holds true within virtual and hybrid teams. Additionally, future studies could investigate which resource gains are most effective in counterbalancing resource losses and the degree of consistency required to achieve a net positive outcome.

Another avenue for future research is to explore the role of emotional contagion in shaping resource dynamics. As highlighted in our findings, emotional contagion appeared to play a significant role in influencing both resource gains and losses. Future studies could analyse the mechanisms of emotional contagion, examining how positive and negative emotional states propagate within teams and their long-term implications for team cohesion and well-being (Zeijen et al., 2020).

Additionally, as hybrid work models continue to evolve, it is important to understand how individuals and teams adapt over time. Longitudinal studies following the same cohort of participants would provide invaluable insights into the long-term effectiveness of well-being practices, as well as the emergence of new demands and resources as hybrid work practices mature. Such research could inform the development of adaptive organizational strategies that evolve in tandem with changes in work environments and team needs.

Finally, future research would benefit from a more diverse participant pool, encompassing a broader range of industries, geographic locations, and demographic groups. This would enable a more inclusive understanding of how cultural, sectoral, and individual differences shape well-being experiences in virtual and hybrid teams. Furthermore, future studies could examine how hybrid working affects specific subgroups, such as team members with caregiving responsibilities, those with disabilities, or individuals from underrepresented communities, who may encounter unique demands and resources.

## Conclusion

This study highlighted the significance of adopting a holistic and multi-dimensional approach to fostering team well-being, providing a robust foundation for future longitudinal research. Our findings illustrated the varied and complex pathways teams navigate, which shape well-being outcomes in diverse and sometimes uneven ways. Given this inherent complexity, we advocate for deliberate, proactive practical strategies to cultivate healthier team environments. Such efforts require a blend of short-term interventions and long-term structural initiatives, as neglecting these aspects could undermine both immediate and sustained team well-being.

By extending the JD-R framework (Bakker & Demerouti, 2007) and incorporating insights from Conservation of Resources (COR) theory, we have illuminated the dynamic, context-sensitive interaction of demands and resources. This demonstrated the pivotal role of intentional team practices and leadership behaviours in promoting long-term, sustainable well-being. Grounded in the lived experiences of team members and leaders, our findings offer a pragmatic roadmap for organizations aiming to develop healthier and more resilient virtual and hybrid teams. We call for a strategic reorientation of well-being drivers, positioning them as core elements of organizational culture. By embedding this integrated approach, organizations can create an environment that not only elevates workforce well-being and engagement but also enhances long-term organizational resilience, recognizing that their people are the most vital asset in an ever-evolving world of work.

## Supplementary Material

Final Indicative question guide for team leader.docx

Final Indicative question guide for focus groups.docx

Author_Biographies .docx
