# Supplementary material for: Advancing virtual and hybrid team well-being through a job demand-resources lens
Source: Int J Qual Stud Health Well-being. 2025 Mar 13;20(1):2472460. doi: 10.1080/17482631.2025.2472460 (PMC11916424; doi:10.1080/17482631.2025.2472460)
Supplement: Final Indicative question guide for team leader.docx [file ZQHW_A_2472460_SM9222.docx]

**Indicative questions for 60-minute semi-structured team leader interview**

**Introduction (5 mins)**

I am a researcher working with King’s College London in the Institute of Psychiatry, Psychology and Neuroscience. The purpose of this study is to explore your views relating to virtual or hybrid working as a team leader. Specifically, we aim to understand some of the key factors that may help increase your levels of well-being and performance, as well as factors that hinder your wellbeing and performance.

Can I check you have read the information sheet that has been circulated and that you are consenting to take part in this interview? All will be anonymously transcribed within 10 days and the recording destroyed. Any questions?

A reminder that all the questions are voluntary, so if at any time, you would prefer not to answer, that is completely fine.

Context driven questions: **What does your working context look like right now?**

**Section 1: (30 mins)**

**Understanding factors that impact well-being outcomes of the team and team leader**

- **What does work-related well-being mean to you?**
- **What about team well-being?**
- **Could you share any examples of well-being practices you have a team that help your levels of well-being?** *Any specific rituals or meetings? What is the most important? Anything that gets in the way of you doing this as a team?*
- What else may help improve your levels of well-being as a team? No meetings days? Videos on or off? Celebrating team successes? Utilising strengths?

*Are there any reasons these aren’t being used now/barriers to implementation?*

- **What would you say are some of the biggest demands you face as a leader that can lead to stress?**

*How empowered do you feel to take action on this? What support could you benefit from?*

- In what ways do you communicate as a leader with your team? For example, how often do you have video meetings? E-mail? Whatsapp? Phone calls?
- As a team do you have any ‘norms’ of communication?
- ***How important is how you communicate as a team to your well-being?* How do you communicate most as a team? *As a leader?*** *Shared norms? What does ‘good’ virtual communication look like? Do you experience this regularly as a team?*
- How important are levels of trust in your team-members to your personal wellbeing?

*How would you describe ‘trust’ within your team? What does a good level of ‘trust’ look like? Anything that gets in the way of developing this?*

- ***In a virtual or hybrid team environment, how do you lead? Are there differences to how you would lead in person teams? If so, what?*** *How important is shared leadership as a team?*

*What are the biggest differences? What is the same? How challenging is this for you?*

**Section 2: (25 mins)**

**Understanding factors that impact performance outcomes of the team at an individual and collective level**

- **What are some of the factors that help your performance as a team? What about as a leader?** *How important is having a shared vision? What about feeling safe enough to share thoughts and feelings? How important is trust? What kind of trust is important? Are any other behaviours or processes important?*
- **Are there factors that hinder your performance as a leader/as a team? Which are the most influential and why?**

*Anything else? How empowered do you feel to be able to act on this? What would help?*

- How important is your team’s collective performance to your own individual performance? *What can you not achieve without your team?*

**Concluding statements and thoughts**

- This brings us almost to the close of this focus group. I’m wondering if there are any other areas that you feel have not been addressed that you would like to share your views on?
- Anything else that has not been said that anyone would like to add?

NB: For all the above, will include additional probing questions, where relevant:

Tell me more…

What would that look like?

If you were being even more specific on that, what would you say?

What would the opposite of that look like?

NB: Questions in bold are the ‘must have’ questions to be asked in every inquiry.
