# Supplementary material for: Advancing virtual and hybrid team well-being through a job demand-resources lens
Source: Int J Qual Stud Health Well-being. 2025 Mar 13;20(1):2472460. doi: 10.1080/17482631.2025.2472460 (PMC11916424; doi:10.1080/17482631.2025.2472460)
Supplement: Final Indicative question guide for focus groups.docx [file ZQHW_A_2472460_SM9221.docx]

**Indicative questions for 60-minute qualitative focus groups**

**Introduction (5 mins)**

I am a researcher working with King’s College London in the Institute of Psychiatry, Psychology and Neuroscience. The purpose of this study is to explore your views relating to virtual or hybrid working as part of a team. Specifically, we aim to understand some of the key factors that may help increase levels of well-being and performance, as well as factors that hinder well-being and performance.

Can I check everyone has read the information sheet that has been circulated and that you are consenting to take part in this focus group? All will be anonymously transcribed within 10 days of this focus group and the recording destroyed. Does anybody have any questions at this stage?

Is everybody happy to proceed with the discussion?

Context driven questions: **What does your working context look like right now?**

**Section 1: (30 mins)**

**Understanding factors that impact well-being outcomes of the team (individual & collective)**

- **What does team well-being mean to you?**
- **Could you share any examples of well-being practises you have a team that help your levels of well-being?** *Any specific rituals or meetings? What is the most important? Anything that gets in the way?*
- What else may help improve your levels of well-being as a team? No meetings days? Videos on or off? Celebrating team successes? Utilising strengths?

*Are there any reasons these aren’t being used now/barriers to implementation?*

- **What would you say are some of the biggest demands you face as a team that can lead to stress?**

*How empowered do you feel to take action on this? What support could you benefit from?*

- In what ways do you communicate as a team?
- As a team do you have any ‘norms’ of communication?
- ***How important is how you communicate as a team to your well-being? How do you communicate most as a team?*** *Shared norms? What does ‘good’ virtual communication look like? Do you experience this regularly as a team?*
- How important are levels of trust in your team-members to your personal well-being?

*How would you describe ‘trust’ within your team? What does a good level of ‘trust’ look like? Anything that gets in the way of developing this?*

**Section 2: (25 mins)**

**Understanding factors that impact performance outcomes of the team at an individual and collective level**

- What is important for effective virtual/hybrid ‘teamwork’? Norms of how you communicate. Levels of trust? Knowledge sharing?
- **What are some of the factors that help your performance as a team?** *How important is having a shared vision? What about feeling safe enough to share thoughts and feelings? How important is trust? What kind of trust is important? Are any other behaviours or processes important?*
- **Are there factors that hinder your performance as a team? Which are the most influential and why?**

*Anything else? How empowered do you feel to be able to act on this? What would help?*

- How would you describe how you are led as a team by your team-leader?

*What, if anything, would you like them to do differently?*

- **In a virtual or hybrid team environment, how would you like to be led?** *Are there differences to in person teams? If so, what? How important is shared leadership as a team?*

*What are the biggest differences? What is the same? How challenging is this for leaders?*

**Concluding statements and thoughts**

- This brings us almost to the close of this focus group. I’m wondering if there are any other areas that you feel have not been addressed that you would like to share your views on?
- Anything else that has not been said that anyone would like to add?

NB: For all the above, will include additional probing questions, where relevant:

Tell me more…

What would that look like?

If you were being even more specific on that, what would you say?

What would the opposite of that look like?

NB: Questions in bold are the ‘must have’ questions to be asked in every inquiry.
