# Supplementary material for: Advancing virtual and hybrid team well-being through a job demand-resources lens
Source: Int J Qual Stud Health Well-being. 2025 Mar 13;20(1):2472460. doi: 10.1080/17482631.2025.2472460 (PMC11916424; doi:10.1080/17482631.2025.2472460)
Supplement: Author_Biographies .docx [file ZQHW_A_2472460_SM9220.docx]

**Professor Sukhi Shergill**

Sukhi Shergill is Professor of Psychiatry at KMMS, Consultant Psychiatrist and Director of Research at the Kent wide NHS trust (KMPT) since October 2021. He is also Professor of Psychiatry and Systems Neuroscience at the Institute of Psychiatry, Psychology and Neuroscience, King’s College London (IoPPN-KCL). He trained in medicine at University College London (UCL), completing a BSc in Psychology before starting his psychiatry training at UCL and completing his higher training at the Maudsley Hospital London. He obtained his PhD at the Institute of Psychiatry KCL and was the recipient of several research fellowships before promotion to Professor in 2014.

Sukhi heads the Cognition, Schizophrenia and Imaging Laboratory (CSI-Lab), comprising two-dozen academics, clinical researchers, PhD and other students. He was the founding director of the King’s centre for innovative therapies developing novel therapeutic approaches to mental health between industry and academics/clinicians within KCL.

Sukhi has been the recipient of several research and teaching awards; has had extensive grant funding from a range of governmental and charitable sources; published 250+ peer reviewed research publications and supervised more than 20 PhD students.

**Dr Myanna Duncan**

Dr Myanna Duncan is a Senior Lecturer (Associate Professor) in Occupational Psychology at the Institute of Psychiatry, Psychology & Neuroscience (IoPPN) at King's College London. Myanna is a Chartered Occupational Psychologist (British Psychological Society) with a wide range of industry and academic experience, specialising in Occupational Health Psychology. She joined King’s in 2014 where she worked at the Florence Nightingale Faculty of Nursing, Midwifery & Palliative Care, prior to moving to the IoPPN in 2016.Prior to this, Myanna worked at the National Institute of Heath Research (NIHR) Biomedical Research Centre, Loughborough University.

Myanna holds a PhD in Applied Psychology from Loughborough University, an MSc. from The University of Nottingham and a BSc. from the University of Warwick. She has secured funding from The Wellcome Trust, BBSRC, Deloitte LLP and the ESRC.

Myanna reviews for several key journals in her field and is an external examiner at the University of Derby for the MSc in Behaviour Change programme.

**Dr Ricardo Twumasi**

Ricardo Twumasi is a lecturer at King’s College London within the Institute of Psychiatry, Psychology and Neuroscience. As a principal investigator, he has led research initiatives encompassing resilience in healthcare workers, health promotion, workplace discrimination, and established an anti-racist health inequalities journal. His research interests also include equality, age discrimination, gerontology, behavioural change, workplace health promotion, artificial intelligence, and machine learning.

**Cass Coulston**

Cass Coulston is a PhD researcher at King’s College London, which she combines with extensive working experience in consulting, leadership and team coaching.

As a previous Commercial Director and General Manager of global businesses, she has also lived in Hyderabad, India, for five years, where she designed and delivered the leadership and resilience coaching programmes for leaders and teams in global organisations. Cass graduated from Birkbeck, University of London with a Distinction in Organisational Psychology MSc in 2019 and won awards for best research project and best performer award.

Cass’s PhD study is co-funded by Deloitte, a global professional services firm and the London Interdisciplinary Social Science Doctoral Training Partnership (LISS DTP). Her study aims to identify the psychological, social and environmental variables, that influence well-being, mental health and performance outcomes of virtual and hybrid teams. The key factors considered amenable to change will then be tested in an intervention study examining well-being and performance outcomes at an individual and team level.

Cass is also passionate about teaching and supports the delivery of the BSc Psychology programme by giving a guest lecture series on the Work Psychology module
